# Allergy and sensitization to Hymenoptera venoms in unreferred adults with a high risk of sting exposure

**DOI:** 10.1016/j.waojou.2019.100039

**Published:** 2019-06-28

**Authors:** Alexander Zink, Barbara Schuster, Julia Winkler, Kilian Eyerich, Ulf Darsow, Knut Brockow, Bernadette Eberlein, Tilo Biedermann

**Affiliations:** Department of Dermatology and Allergy, Technical University of Munich, Munich, Germany

**Keywords:** Anaphylaxis, Hymenoptera venom sensitization, Outdoor population, Recombinant allergens, Sting reaction

## Abstract

**Background:**

Hymenoptera venom sensitization in highly exposed individuals frequently requires risk assessment for future severe sting reactions. In this study, we determined the prevalence of Hymenoptera venom sensitization in individuals who hunt and fish and analyzed possible correlations between the severity of sting reactions and the IgE sensitization profile.

**Methods:**

In this cross-sectional study, paper-based, self-filled questionnaires about previous insect stings and sting reactions were obtained from individuals who hunt and fish in Bavaria, Germany. Blood samples were taken and analyzed for the levels of tryptase, total IgE and IgE to honey bee (i1) and wasp (13) venom, the recombinant allergens rApi m 1, rApi m 2, rApi m 3, rApi m 5, rApi m 10, rVes v 1, rVes v 5, and the CCD marker molecule MUXF3. Odd ratios (ORs) for sensitization and anaphylaxis and Pearson's correlations for the different allergens were calculated.

**Results:**

Of 257 participants, 50.2% showed a sensitization to honey bee venom (i1), and 58.4% showed sensitization to wasp venom (i3). A total of 98.4% of participants claimed to have been stung at least once. Anaphylaxis was reported in 18.7%, and a local sting reaction was reported in 18.3%. The highest sensitization rates were found for whole venom extracts, sensitization to any of the available recombinant allergens exceeded sIgE levels to honeybee venom (i1) in 28.5% and to wasp venom (i3) in 52.9% of participants. Participants with a history of more than 5 stings showed a higher risk for anaphylaxis.

**Conclusions:**

Sensitization to Hymenoptera venom and their recombinant allergens are present in the majority of individuals who hunt and fish. Sensitization to distinct recombinant allergens does not necessarily affect the severity of sting reactions including anaphylaxis. A meticulous medical history of the number of previous stings as well as systemic reactions remains essential.

## Introduction

Insect stings and sensitization to Hymenoptera venom are common in the general population.^1^, ^2^, ^3^, ^4^, ^5^ Although different methods used for assessing sensitization make it difficult to compare different populations, sting frequency and sensitization rates in warm, southern countries seem to be higher than in countries with a cooler climate^3^, ^4^: Studies from Sweden^3^ and Denmark^1^ showed sensitization rates of 9% and 15% respectively, while a study from Turkey^4^ reported that 29% of the general population are sensitized. Similarly, individuals who spend a lot of time outside, such as those who hunt and fish, are prone to repeated stings and therefore have a very high risk of sensitization and venom allergy.^6^, ^7^, ^8^ However, there are few data on the prevalence of sensitization and allergy in these population groups.

A previous study that determined the risk of systemic sting reactions in individuals with “hitherto irrelevant sensitization” suggested that “sensitization to Hymenoptera venoms is common, but systemic sting reactions are rare".^2^ Clinically irrelevant sensitization has been shown to be related to high total IgE levels^9^ while severe sting reactions are associated to high tryptase levels.^10^, ^11^, ^12^, ^13^ However, discrimination between asymptomatic and potentially clinically relevant sensitization is still not possible without a history of previous reactions. In addition, there is no known parameter that allows the forecast of the severity and risk in regard to future allergic reactions to Hymenoptera venoms based on sensitization profiles.

Immunotherapy is a highly effective treatment to prevent severe systemic reactions, but time-consuming and expensive.^14^, ^15^, ^16^ Therefore, it is only performed in patients with previous systemic allergic reactions, even though this treatment could also prevent systemic allergic reactions in individuals sensitized and prone to react. Optimized *in vitro* assessments of the risk for severe allergic reactions would be highly appreciated and useful for doctors as well as patients. Specific IgE (sIgE) to different single allergens, such as rApi m 1, rApi m 2, rApi m 3, rApi m 5 and rApi m 10 (for honey bee venom), rVes v 1 and rVes v 5 (for wasp venom), have increasingly gained diagnostic importance in addition to the common honey bee venom (i1) and wasp venom extracts (i3).^17^ Recombinant allergens show diagnostic advantages in sensitivity and specificity, especially concerning the problem of cross-reactivity.^17^, ^18^, ^19^ However, to date, the sensitization profile to different recombinant allergens cannot be used to forecast the risk of a sensitized individual.

In contrast, different sensitization profiles in peanut or peach allergy are associated with different forms of allergic reactions. Studies in southern Europe showed that patients with peach allergy showing high sIgE-levels to Pru p 3 are more likely to show systemic reactions.^20^, ^21^ For peanut allergy, elevated sIgE to Ara h 1, 2 and 3 have been found to increase the risk for severe allergic reactions.^22^, ^23^ These findings permit a better risk assessment for patients suffering from peanut or peach allergy by their medical doctors. Analogous findings would be invaluable for Hymenoptera venom allergy.

In our study, we aimed to investigate the prevalence of bee and wasp sensitization in a high-risk population for insect stings, to analyze the sensitization profile and to investigate potential correlations between symptoms (anaphylaxis grade 1 to 4 and pronounced local reaction to sting) and sensitization to different recombinant allergens.

## Methods

### Subjects

This cross-sectional study was performed at the annual winter meetings of three different hunting associations (Wolfratshausen, Landsberg am Lech and Freising) in December 2016 and at an annually held international exhibition for hunting and fishing (“Jagen und Fischen Augsburg”) in January 2017, all located in the Greater Munich Area, Southern Germany. The study was approved by the local ethics committee of the Medical Faculty of the Technical University of Munich (405/15s), and written informed consent was obtained from all participants prior to study inclusion. All participants had to be individuals 18 years or older who actively hunted or fished. Exclusion criterion was the inability to understand the study information and/or the paper-based study questionnaire due to language barriers or other circumstances. Subjects included in the study first had to fill out a paper-based questionnaire before blood samples were obtained and frozen for later *in vitro* tests.

### Self-reported questionnaire

Completion of the paper-based questionnaire was a prerequisite for the blood withdrawal (performed by medical doctors). Apart from general data (age, sex and residence) and profession/hobby (hunting, fishing), the paper-based questionnaire included questions on known allergies (pollen, food, drugs, other), number of previous insect stings by Hymenoptera (0, 1, 2–5, more than 5 stings) and any experienced local or generalized reactions after Hymenoptera stings. Only the total number of stings by any Hymenoptera (honeybee, wasp, bumblebee and hornet) was counted, not distinguishing between species, to minimize memory bias. In this European study, reactions to insect stings were assessed with questions on local reactions and questions according to the European Grading of Anaphylactic Symptoms According to Severity of Symptoms,^24^, ^25^ allowing a classification from anaphylaxis grade 1 to grade 4:

- Local reactions: erythema, pain, edema
- Generalized reactions:
  ○ Dermal (pruritus, flushing, urticaria, angioedema)
  ○ Abdominal (nausea, cramping, vomiting, defecation, diarrhea)
  ○ Respiratory (rhinorrhea, hoarseness, dyspnea, laryngeal edema, bronchospasm, cyanosis, respiratory arrest)
  ○ Cardiovascular (tachycardia, blood pressure change, arrhythmia, shock, cardiac arrest)

### In vitro tests

We used the ImmunoCAP system (Thermo Fischer Scientific/Phadia, Uppsala, Sweden) according to the instructions of the manufacturer in order to determine total and specific IgE. Allergen-specific IgE positivity was determined using both a cutoff of ≥0.35 kU/l, which is typically used in clinical allergy, and of ≥0.1 kU/l, which is applied by the manufacturer of the ImmunoCAP assay and which has been proven to provide a higher sensitivity.^26^ Total IgE levels were classified in 3 groups (<20, 20–100, >100 kU/l). Serum tryptase was measured with an ImmunoCAP Tryptase assay, for which 11.4 μg/l has been adopted as the upper reference level in previous studies.^27^

Blood samples were obtained and stored frozen until tested. After unfreezing, all sera were tested for tryptase, total IgE and specific IgE to honey bee (*A. mellifera)* venom (ImmunoCap code i1) and the corresponding recombinant major allergens rApi m 1 (i208), rApi m 2 (i214), rApi m 3 (i215), rApi m 5 (i2016) and rApi m 10 (i217), as well as to venom from wasp species (*V. vulgaris, V. germanica)* (i3) and the recombinant major allergens rVes v 1 (i211), rVes v 5 (i209). Furthermore, specific IgE to the isolated glycan part from bromelain (o214) was measured to assess reactivity to Cross-reactive Carbohydrate Determinant (CCD) (MUXF3 CCD) to screen for clinically irrelevant sensitizations also responsible for serologic cross-reactivity between bee and wasp venom.

### Statistics

Data were analyzed using SPSS statistics 23.0 (IBM Corp.). In addition to descriptive parameters, *P* values of Chi-square tests or odds ratios (OR) and 95% confidence intervals (CI) are given as parameters of association and stability. Pearson's correlations for the specific IgE levels were calculated to appraise if the number of measures of different recombinant allergens can be reduced and simplified.

## Results

### Self-reported questionnaire

A total of 257 individuals who actively hunt and fish (40 women, 217 men) participated in the study. Mean age was 50.6 ​± ​14.7 years (range 18–83 years).

#### Self-reported previous stings

Of all 257 participants, 4 (1.6%) did not remember any previous Hymenoptera sting, whereas 19 (7.4%) reported that they remembered a single previous sting, 59 (23.0%) reported 2 to 5 stings and 163 (63.4%) reported more than 5 previous stings. Twelve participants (4.7%) did not answer the question. In general, women reported less previous stings than men. While 7.7% of women did not remember a previous Hymenoptera sting, and 46.2% reported more than 5 stings, only 1 man (0.5%) did not remember any previous stings, and 70.4% of men reported more than 5 stings.

#### Self-reported allergies

Of all 257 participants, 168 (65.4%) reported that they did not have “any allergies”, and 85 (33.1%) claimed that they suffered from an allergy (pollen, food, drugs, Hymenoptera venom, etc.). A total of 91.8% of the participants did not state “allergy against Hymenoptera venom”, 4 participants (1.6%) did not give an answer, and 17 (6.6%) claimed to suffer from an Hymenoptera venom allergy, including honey bee venom 8 (3.1%), wasp venom 4 (1.6%) or combined honey bee venom and wasp venom allergy 5 (1.9%). In contrast to these claims, 48 participants (18.7%) reported symptoms consistent with anaphylaxis after Hymenoptera stings. Accordingly, the following local and generalized reactions after Hymenoptera stings were reported in the paper-based questionnaire:

- No anaphylactic reaction: 198 of 257 (77.0%)
  ○ Local reaction only (erythema, pain, edema): 47 of 257 (18.3%)
- Most severe generalized reaction: 48 of 257 (18.7%)
  ○ Anaphylaxis Grade 1: 16 of 257 (6.4%)
  ○ Anaphylaxis Grade 2: 8 of 257 (3.2%)
  ○ Anaphylaxis Grade 3: 12 of 257 (4.8%)
  ○ Anaphylaxis Grade 4: 12 of 257 (4.8%)

#### Previous stings and anaphylactic reactions

Table 1 shows the percentage of participants with less than 2, 2 to 5 and more than 5 previous Hymenoptera stings that reported symptoms consistent with anaphylactic reactions and local reactions only to Hymenoptera venom. Only 1 participant (5.3%) with less than 2 stings reported anaphylactic reactions to a sting. In contrast, 37 (22.7%) of the participants with more than 5 previous stings reported anaphylactic reactions. While the percentage of participants with anaphylactic reactions to Hymenoptera venom increased with the number of previous stings, the percentage of participants with only local reactions decreased. The prevalence of reported anaphylactic reactions to Hymenoptera stings was higher in participants with more than 5 stings compared to participants with 5 or fewer stings (22.7% vs. 12.8%, *Pχ2* ​= ​0.049). Accordingly, participants with more than 5 stings had a higher chance of an anaphylactic reaction to previous Hymenoptera stings.

**Table 1:** Reported anaphylactic reactions after Hymenoptera sting and reported number of previous Hymenoptera stings.

Previous Stings	No anaphylactic reaction		Anaphylaxis				
	Local reaction only (n ​= ​46)	total (n ​= ​198)	Grade 1 (n ​= ​16)	Grade 2 (n ​= ​8)	Grade 3 (n ​= ​11)	Grade 4 (n ​= ​12)	Total (n ​= ​47)
<2 (n ​= ​19)	26.3% (5)	94.7% (18)	–	5.3% (1)	–	–	5.3% (1)
2-5 (n ​= ​59)	20.3% (12)	84.7% (50)	5.1% (3)	1.7% (1)	5.1% (3)	3.2% (2)	18.6% (9)
>5 (n ​= ​163)	17.8% (29)	77.3% (126)	8% (13)	3.7% (6)	4.9% (8)	6.1% (10)	22.7% (37)

Anaphylactic reactions and number of previous Hymenoptera stings; N ​= ​241 (12 did not answer question on number of previous stings, 4 excluded because they reported no previous stings and consequently no reactions).

### In vitro data

#### Total IgE and tryptase levels

Mean total Immunoglobulin E (IgE) measured in 257 sera was 118.44 ​± ​247.64 kU/l (Median 43.3 kU/l; range 0.01 kU/l to 2615 kU/l). Classified into the 3 groups typically used in clinical settings, a total IgE level <20 kU/l was measured in 82 (31.9%) of all 257 sera, a total IgE level of 20–100 kU/l in 109 (42.4%) sera and a total IgE level >100 kU/l in 66 (25.7%) sera. The mean tryptase level in all sera was 3.74 ​± ​3.48 ​μg/l. Normal tryptase levels, which are below 11.4 μg/l according to the manufacturer,^27^ were detected in 248 (96.5%) of all cases, whereas 9 (3.1%) participants showed an elevated basal tryptase level ranging from 11.4 ​μg/l to 36.0 ​μg/l. Of these 9 participants, 1 had a history of systemic reactions to Hymenoptera stings.

#### Hymenoptera venom sensitization

**Fig. 1:**
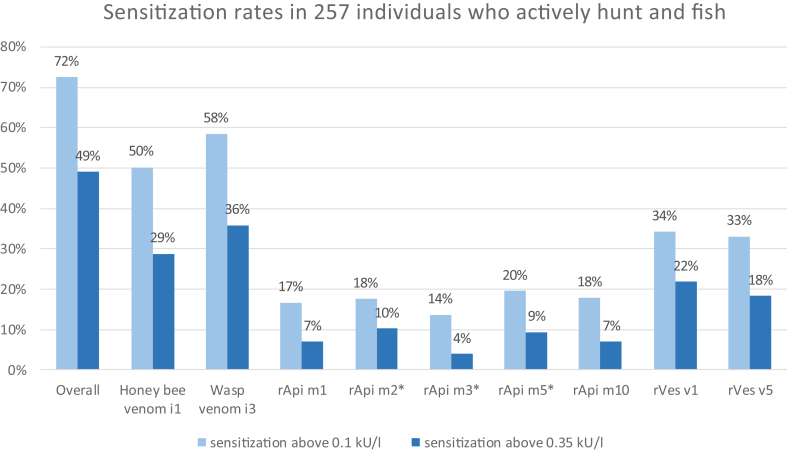
Sensitization rates against honey bee (i1) and wasp (i3) venom as well as recombinant allergens of honeybee venom (rApi m1, rApi m2, rApi m3, rApi m5, rApi m10) and wasp venom (rVes v1, rVes v5) in 257 individuals who hunt and fish. N=257. *one missing case, N=256.

Independent of any clinical symptoms, 129 (50.2%) of all 257 participants showed a sensitization to honey bee venom (i1) ≥0.1 kU/l, and 75 (28.8%) ≥0.35 kU/l (Fig. 1). A total of 150 (58.4%) participants showed a sensitization to wasp venom (i5) with a specific IgE level ≥0.1 kU/l, and 92 (35.8%) ≥0.35 kU/l. In terms of recombinant allergens, highest sensitization rates were seen for rVes v 1, and the lowest rates were found for rApi m 3. Specific IgE antibodies against CCD MUX F3 were found to be ≥0.1 kU/l in 15.2% of all cases and ≥0.35 kU/l in 6.6% of cases. Table 2 displays the mean, standard deviation, median and range of specific IgE antibodies against honey bee and wasp venom and their recombinant allergens. Table 3 displays the percentages of participants sensitized only to honey bee venom or wasp venom respectively and the percentage of double-sensitizations. Participants that were sensitized to honey bee venom (i1) had a higher chance of sensitization to wasp venom (i3) and vice versa (≥0.1 kU/l: OR ​= ​4.29, CI ​= ​[2.52; 7.3]; ≥0.35 kU/l: OR ​= ​3.79, CI ​= ​[2.15; 6.68]). However, 12.45% of all participants were sensitized ≥0.1 kU/l to honey bee venom (i1) but not to wasp venom (i3) and 20.62% were sensitized ≥0.1 kU/l to wasp venom (i3) but not to honey bee venom (i1) (Table 3). For sensitization ≥0.35 kU/l, this was the case for 12.1% and 19.1% of all participants, respectively. Regarding honey bee venom (i1) and its recombinant allergens, 28.5% of all participants showed a higher specific IgE level for sensitization to the recombinant allergens (all specific IgE levels of the recombinant allergens added together) than for sensitization to the extract as a whole. For wasp venom (i3) and its recombinant allergens, almost twice as many participants (52.9%) showed a higher specific IgE level for sensitization to the recombinant allergens than for sensitization to the extract as a whole. Regarding honey bee venom and its recombinant allergens, 3.5% of all participants showed a sensitization ≥0.1 kU/l and 2% above ≥0.35 kU/l to at least one of the recombinant allergens but not to the whole extract. For wasp venom, 2 participants (0,8%) showed a sensitization ≥0.1 kU/l and 1.6% of all participants ≥0.35 kU/l to at least 1 of the recombinant allergens but not to the whole extract. With regard to gender, more male (72.4%) than female (62.5%) participants were tested positive for a sensitization to honey bee or wasp venom.

**Table 2:** Mean, standard deviation (SD), median and range (minimum, maximum) of levels of specific IgE antibodies against honey bee (i1) and wasp (i3) venom as well as recombinant allergens of honey bee venom and wasp venom and CCD MUX F3 in 257 participants.

Allergen	Mean kU/l	SD kU/l	Median kU/l	Min kU/l	Max kU/l
Honey bee i1	0.95	3.92	0.1	0	48.3
Wasp i3	1.01	3.1	0.18	0	29.1
rApi m1	0.17	1.03	0	0	13.9
rApi m2^a^	0.25	1.55	0.1	0	23.1
rApi m3^a^	0.06	0.20	0	0	1.79
rApi m5^a^	0.3	2.59	0	0	40.4
rApi m10	0.23	1.84	0.01	0	29
rVes v1	0.45	1.41	0.04	0	13.3
rVes v5	0.39	1.69	0.04	0	22.1
CCD MUX F3	0.22	1.45	0	0	20.7

Mean, standard deviation (SD), median and range (minimum, maximum) of levels of specific IgE antibodies against honey bee (i1) and wasp (i3) venom as well as recombinant allergens of honey bee venom and wasp venom and CCD MUX F3 in individuals who hunt and fish. N ​= ​257.

a One missing case, N ​= ​256.

**Table 3:** Sensitization rates in individuals who hunt and fish (N ​= ​257) to honey bee venom (i1) and wasp venom (i3) and their respective recombinant allergens, including double-sensitization.

Species	Sensitization to …					
	… the whole extract		… at least one of the recombinant allergens^a^		… the whole extract or at least one of the recombinant allergens^a^	
	≥0.1 kU/l	≥0.35 kU/l	≥0.1 kU/l	≥0.35 kU/l	≥0.1 kU/l	≥0.35 kU/l
Honey bee only	12.5%	12.1%	14.5%	11.3%	13.3%	11.7%
Wasp only	20.6%	19.1%	23%	21.9%	18.8%	18.4%
Honey bee and wasp	37.7%	16.7%	26.6%	9%	40.6%	19.1%

Sensitization rates to honey bee venom (i1) and wasp venom (i3) and their respective recombinant allergens including double-sensitization. N ​= ​257.

a One missing case for rApi m2, rApi m3 and rApi m5.

The analysis with respect to age categories (18–30, 31–40, 41–50, 51–60 and 61–70, >70 years) revealed no interconnection between age and sensitization for sIgE ≥0.1 kU/l (honeybee: *Pχ2* ​= ​0.62; wasp: *Pχ2* ​= ​0.697) or sIgE ≥0.35 kU/l (honeybee: *Pχ2* ​= ​0.391; wasp: *Pχ2* ​= ​0.626).

As previously mentioned, 25.7% of all subjects (and 29.3% of subjects with a clinically relevant sensitization) had elevated total IgE levels >100 kU/l. All subjects with total IgE levels >100 kU/l showed a strikingly higher frequency of sensitization than those with total IgE levels <20 kU/l (≥0.1 kU/l: OR ​= ​14.06; ≥0.35 kU/l: OR ​= ​10.79) and with total IgE levels 20–100 kU/l (≥0.1 kU/l: OR ​= ​6.44; ≥0.35 kU/l: OR ​= ​4.38). However, clinically relevant sensitization to Hymenoptera venom was not significantly more common in participants with total IgE levels >100 kU/l than in those with total IgE levels <20 kU/l.

**Fig. 2:**
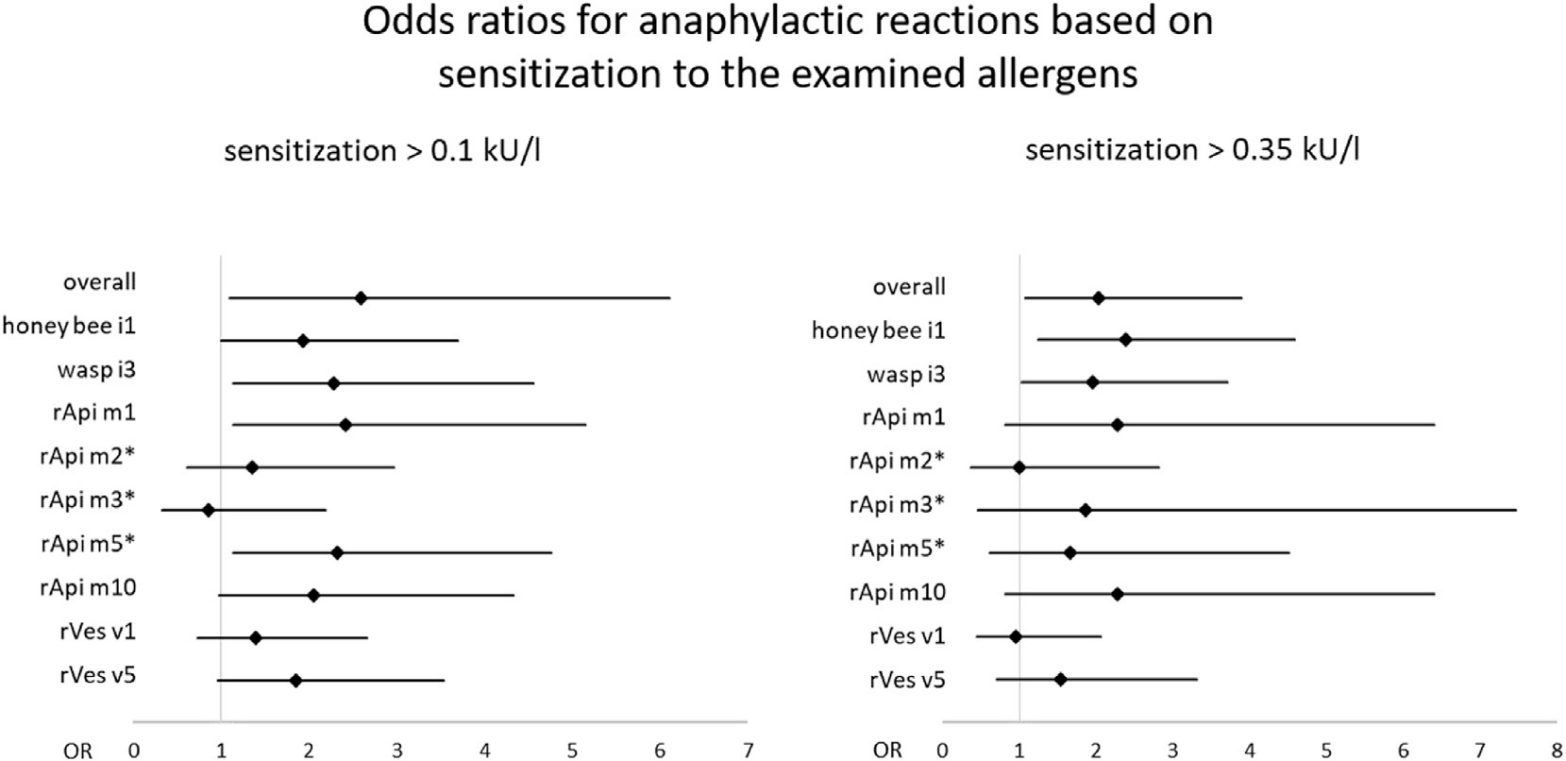
Odds Ratios and confidence intervals for anaphylactic reactions based on sensitization to the examined allergens. Reference group sIgE < 0.1 kU/l and sIgE < 0.35, respectively. N= 257. *one missing case, N=256.

Table 4 shows sensitization rates (≥0.1 kU/l and ≥0.35 k/UL) against honey bee (i1) and wasp (i3) venom as well as to recombinant allergens of participants reporting anaphylactic reactions and local reactions. A sensitization ≥0.1 kU/l to at least 1 of the examined allergens was found in 85.4% of those who had a history of systemic reactions to Hymenoptera stings, in contrast to sensitization rates of 69.3% in those without a history (OR ​= ​2.59, CI ​= ​[1.1; 6.1], Fig. 2). For sensitization ≥0.35 kU/l the rates were 62.5% and 45% (OR ​= ​2.03, CI ​= ​[1.07; 3.89]). Odds ratios and confidence intervals for the sensitization to the different allergens of participants with a history of anaphylactic reactions are displayed in Fig. 2. Participants with clinically relevant allergy did not show higher levels of relative sIgE (level of sIgE divided by the total IgE level) for any of the examined allergens compared to participants with sensitizations but no history of systemic sting reactions (considering both >0.01 kU/l and >0.35 kU/l).

**Table 4:** Sensitization rates for honey bee and wasp venom sensitization and their recombinant allergens and MUX F3, shown separately for participants with and without reported anaphylactic reactions, in percent (N ​= ​257).

Specific IgE in kU/l		Anaphylaxis (n ​= ​48)					No anaphylactic reaction (n ​= ​203)		No information provided on anaphylaxis (n ​= ​6)^a^
		Grade 1 (n ​= ​16)	Grade 2 (n ​= ​8)	Grade 3 (n ​= ​12)	Grade 4 (n ​= ​12)	Grade 1–4 (n ​= ​48)	Total (n ​= ​203)	Local reaction only (n ​= ​47)	
honey bee i1	≥0.1	56	63	58	75	63	46	62	83
	≥0.35	31	38	50	58	44	25	34	50
wasp i3	≥0.1	56	88	75	83	73	54	62	83
	≥0.35	38	63	42	58	48	32	36	67
rApi m1	≥0.1	19	0	42	42	27	13	23	33
	≥0.35	6	0	33	8	13	6	15	0
rApi m2^b^	≥0.1	13	13	25	33	21	16	26	33
	≥0.35	13	0	13	8	10	10	15	0
rApi m3^b^	≥0.1	6	0	8	33	13	14	19	0
	≥0.35	6	0	0	17	6	3	6	0
rApi m5^b^	≥0.1	31	25	42	25	31	16	28	33
	≥0.35	6	0	25	17	13	8	17	33
rApi m10	≥0.1	25	13	13	50	27	15	30	33
	≥0.35	13	0	0	33	13	6	17	0
rVes v1	≥0.1	38	38	50	33	40	32	38	67
	≥0.35	19	38	8	25	21	22	25	33
rVes v5	≥0.1	25	75	42	50	44	30	28	67
	≥0.35	6	38	33	25	23	16	15	50
MUX F3	≥0.1	19	25	13	33	23	14	23	0
	≥0.35	6	13	13	8	10	6	11	0
Total	≥0.1	69	88	92	92	83	67	81	83
	≥0.35	56	63	58	75	63	43	55	83

Sensitization rates for honey bee and wasp venom sensitization and their recombinant allergens and MUX F3, shown separately for participants with and without reported anaphylactic reactions, in percent (N ​= ​257). N ​= ​257.

a Six participants didn't disclose if they have had any reactions to previous Hymenoptera stings.

b One missing case, N ​= ​256.

Participants with more than 5 previous stings more frequently had a clinically relevant sensitization (sensitization and reported anaphylactic reactions) to any of the examined allergens than participants with 5 or fewer stings (≥0.1 kU/l: 22.2% vs. 8.5% with clinically relevant sensitization, OR ​= ​2.72, CI ​= ​[1.147; 6.451]; ≥0.35 kU/l; 14.7% vs. 6.1% with clinically relevant sensitization, OR ​= ​2.659, CI ​= ​[0.975; 7.249]).

#### Correlation analysis

In participants without history of anaphylactic reactions to Hymenoptera venom, most of the sensitizations correlated moderately. Overall, participants with a history of anaphylactic reactions to Hymenoptera stings showed stronger intercorrelations for sensitization to Hymenoptera venoms and their respective recombinant allergens compared to non-anaphylactic participants (Supplementary Table 5).

## Discussion

Our study has shown that individuals who hunt and fish have an elevated risk for insect stings, which we expected. Their cumulative lifetime sting rate in Bavaria, Germany was 98.3%, comparable to a study examining the general population in Turkey (94.5%).^4^ Sensitization against honey bee and wasp venom was found in 50.2% and 58.4%, respectively, of hunting and fishing participants and was higher than in comparable previous studies,^7^, ^28^ that found 40% Hymenoptera venom sensitization in forest workers^7^ and 40% honey bee sensitization in bee keepers.^28^ Similarly, sensitization rates in this high-risk sample were higher compared to a recent study in a German general population-representative cohort, in which the authors found sensitization rates of 23% for honey bee and 32% for wasp venom sensitization.^29^

Anaphylaxis (European grade I-IV) after Hymenoptera stings was reported by 18.7% of all participants. This exceeds previous publications stating percentages ranging from 2% to 7% systemic sting reactions, for example, in Italian forest workers (4.5%),^30^ Spanish (2.3%)^31^ and Bavarian (5.2%)^32^ rural population or suburban population in Denmark (7%).^1^

Interestingly, in contrast to the 18.7% of participants who reported symptoms of anaphylaxis, only 6.6% claimed to have an allergy to Hymenoptera venom. This discrepancy points to a lack of knowledge about Hymenoptera venom allergy in this high risk population group. Considering the risk of potentially severe allergic reactions, it could be reasonable to provide more information about this topic for individuals who hunt and fish (and maybe other highly exposed population groups).

Our study revealed that a history of more than 5 previous insect stings is associated with a higher frequency of sensitization (comparing all tested allergens), and it is a main risk factor for anaphylaxis. Since we could not find this in previous literature, it underlines the importance of a precise assessment of the patients' history regarding previous stings and may be a first risk categorization in more or less than 5 previous stings.

A higher risk of Hymenoptera sensitization in men has been described in several studies and is in accordance with the findings of this study.^3^, ^29^, ^33^ This could be explained by a higher exposure risk for men, who also reported Hymenoptera stings more frequently than women in our study. Although there is some evidence for this hypothesis in the literature,^3^, ^29^, ^33^, ^34^ other studies contradict a gender-related difference^31^.

The significance of the association between atopy and sensitization without clinical relevance is still a matter of debate. Subjects in this study with elevated IgE levels >100 kU/l showed considerably higher rates of sensitization. Studies from Austria, Germany and Sweden found a similar association of sensitization in atopic subjects.^3^, ^9^, ^29^

We consider our outdoor population-based setting to be advantageous to clarify the epidemiology of Hymenoptera venom sensitization and anaphylaxis in populations with numerous and repetitive stings. Available previous epidemiologic studies on Hymenoptera sensitization typically focused on general populations: In a Swedish study from 1993, 9.3% of 1815 randomly chosen subjects showed a sensitization to bee or wasp venom, and 1.5% of all cases reported systemic reactions to bee or wasp stings.^3^ A study from Denmark with 2090 adults revealed a sensitization of 15% to Hymenoptera venom, but only approximately 5% had reacted to stings.^1^ Higher sensitization rates were found in a population-representative study in Germany, where specific IgE antibodies to bee or wasp venom were found in 42% of the population.^29^

Specific IgE levels to whole venom extracts of honey bee (i1) and wasp (i3) venom were higher than sIgE levels to single recombinant allergens. However, when adding sIgE levels for recombinant allergens for honey bee and wasp venom, sIgE to recombinant allergens exceeded sIgE for the whole venom extracts in 28.5% for honey bee and in 52.9% for wasp venom. This finding suggests that measuring sIgE for the combination of all recombinant allergens offers a comparable or even higher sensitivity than exclusively measuring sIgE for the whole venom extracts. This is reinforced by the fact that 3.5% of our participants showed a sensitization above 0.35 kU/l (and 4.3% for 0.1 kU/l) for at least 1 recombinant allergen, but not to the whole venom extracts. The aforementioned method should be considered by all medical professionals, especially in the evaluation of high-risk patients with suspected Hymenoptera venom allergy. Previous studies have already established increased sensitivity to recombinant allergens, especially for rVes v 5 and rVes v 1.^35^, ^36^, ^37^ Consequently, spiking whole wasp venom extracts with recombinant allergens was introduced and used here for sIgE detection with a substantially higher sensitivity.^38^ However, in our study, the sensitivity of spiked whole wasp venom extract was still lower than the sensitivity of Ves v 1 and Ves v 5 taken together. Michel et al^39^ reported that sIgE testing to recombinant allergens of Hymenoptera venom provides better sensitivity in patients with mastocytosis or with otherwise elevated tryptase levels.

The correlation analysis showed that sIgE to almost all recombinant antigens intercorrelate. However, consistent with previous findings,^40^ sIgE to rVes v 5 did not correlate with sIgE to any honey bee venom antigens and therefore seems to be quite specific for true wasp venom sensitization. Intercorrelations of the sIgE to recombinant allergens of honey bee and wasp venom were stronger, respectively, in individuals with a history of systemic sting reactions, while the intercorrelation between sIgE to allergens of the distinct species did not increase. These findings reinforce the hypothesis that component resolved diagnostics (CRD) is a valid tool to detect true sensitization and reduce the problem of cross-reactivity.^19^, ^41^ However, in contrast to previous reports,^42^ we did not find a considerable correlation between sensitization to MUXF3 with the sensitization to bee venom (i1).

Respectively, 14.6% (≥0,1 kU/l) or 37.5% (≥0.35 kU/l) of the cases with a history of anaphylaxis did not show a sensitization to whole venom extracts or recombinant allergens. It is tempting to speculate, that these potentially “hidden sensitizations” could be detected with new recombinant allergens that are still to be developed. The difference of 14.6% and 37.5% depending on the selected threshold level of ≥0.1 kU/l (suggested by the manufacturer of ImmunoCap assay, ThermoFisher Scientific) or ≥0.35 kU/l (typically used in clinical settings) proves the higher sensitivity of the lower threshold which allows the detection of more clinically relevant sensitizations. This advantage was already described by Fischer et al^26^ in a recent publication on type 1 sensitization to alpha-gal and we therefore implemented the 0.1 kU/l threshold in our study.

Due to our high risk population, the results are not fully generalizable. However, the results allow us to conclude that Hymenoptera sensitization is frequent in individuals who hunt and fish and maybe as well in other high-risk groups for insect stings. Interestingly, sensitization to recombinant allergens is common as well, but we were unable to find a correlation between reaction severity and sensitization to any of the hitherto available recombinant allergens unlike Pru p 3 for peach allergy or Ara h 1–3 for peanut allergy. Therefore, to evaluate potential Hymenoptera allergies and distinguish these (for example) from vasovagal reactions after insect stings, a meticulous assessment of the number of previous stings as well as the exact clinical symptoms is still crucial. However, our results reinforce the advantages of CRD for sensitivity in detecting Hymenoptera venom sensitization and discriminatory power for discriminating true double sensitization from cross-reactivity.

## Ethics approval and consent to participate

The study was approved by the local ethics committee of the Medical Faculty of the Technical University of Munich under the reference number 405/15s.

## Consent for publication

Not applicable.

## Availability of data and material

The datasets used and/or analyzed during the current study are available from the corresponding author on reasonable request.

## Competing interests

K. Brockow has received consultation fees from Phadia -Thermo Fisher. T. Biedermann has received consultation fees from ALK Abelló, Sanofi Regeneron, Novartis, Mylan, Phadia – Thermo Fisher. All other authors declare that they have no competing interests.

## Funding

This study was funded by the Department of Dermatology and Allergy of Technical University of Munich and in parts financially supported by ALK-Abello Arzneimittel GmbH.

## Authors' contributions

1. Conception and design of study: AZ, TB.

2. Acquisition of data: AZ, BS, JW.

3. Analysis and interpretation of data: AZ, BS, BE, KE, UD, TB.

4. Drafting manuscript: AZ, BS, JW.

5. Critically revising manuscript for important intellectual content: BE, KE, UD, KB, TB.

6. Final approval of the version to be published: AZ, BS, JW, BE, KE, UD, KB, TB.
