# Supplementary material for: Allergy and sensitization to Hymenoptera venoms in unreferred adults with a high risk of sting exposure
Source: World Allergy Organ J. 2019 Jun 28;12(7):100039. doi: 10.1016/j.waojou.2019.100039 (PMC6610241; doi:10.1016/j.waojou.2019.100039)
Supplement: Multimedia component 1 [file mmc1.docx]

| Supplementary Table 5: Correlation analysis (stratification for participants with and without previous anaphylactic reactions to Hymenoptera stings) | | | | | | | | | | | | | |
| --- | --- | --- | --- | --- | --- | --- | --- | --- | --- | --- | --- | --- | --- |
| Pearson’s Correlation of specific IgE levels of the different allergens in participants with NO previous anaphylactic reaction to Hymenoptera stings (N=203) | | | | | | | | | | | | | |
| allergens | i1 | Api m1 | | Api m2 | | Api m3 | | Api m5 | Api m10 | i3 | Ves v1 | Ves v5 | MUX F3 |
| i1 | 1 |  | |  | |  | |  |  |  |  |  |  |
| Api m1 | .787** | 1 | |  | |  | |  |  |  |  |  |  |
| Api m2^a^ | .851** | .761** | | 1 | |  | |  |  |  |  |  |  |
| Api m3^a^ | .735** | .578** | | .492** | | 1 | |  |  |  |  |  |  |
| Api m5^a^ | .271** | .310 | | .194** | | .239** | | 1 |  |  |  |  |  |
| Api m10 | .836** | .774** | | .934** | | .484** | | .015 | 1 |  |  |  |  |
| i3 | .584** | .196** | | .254** | | .521** | | .558** | .164* | 1 |  |  |  |
| Ves v1 | .469** | .217** | | .375** | | .313** | | .654** | .280** | .778** | 1 |  |  |
| Ves v5 | .165* | .037 | | .045 | | .125 | | .125 | .047 | .532** | .332** | 1 |  |
| MUX F3 | .659** | .288 | | .275** | | .657** | | .371** | .215** | .796** | 381** | .208** | 1 |
| Pearson’s Correlation of specific IgE levels of the different allergens in participants with previous anaphylactic reaction to Hymenoptera stings (N=48) | | | | | | | | | | | | | |
| allergens | i1 | | Api m1 | | Api m2 | | Api m3 | Api m5 | Api m10 | i3 | Ves v1 | Ves v5 | MUX F3 |
| i1 | 1 | |  | |  | |  |  |  |  |  |  |  |
| Api m1 | .969** | | 1 | |  | |  |  |  |  |  |  |  |
| Api m2 | .788** | | .781** | | 1 | |  |  |  |  |  |  |  |
| Api m3 | .822** | | .805** | | .696** | | 1 |  |  |  |  |  |  |
| Api m5 | .336* | | .247 | | .407** | | .32* | 1 |  |  |  |  |  |
| Api m10 | .959** | | .972** | | .768** | | .801** | .274 | 1 |  |  |  |  |
| i3 | .153 | | -.024 | | -.058 | | -.005 | .077 | -.035 | 1 |  |  |  |
| Ves v1 | .186 | | .004 | | -.02 | | -.019 | .098 | -.007 | .936** | 1 |  |  |
| Ves v5 | .112 | | -.046 | | -.73 | | -.018 | .044 | -.065 | .98** | .879** | 1 |  |
| MUX F3 | .259 | | .051 | | -.045 | | .081 | .28 | .072 | .829** | .865** | .762** | 1 |
| Pearson’s correlations of specific IgE levels to honey bee (i1) and wasp (i3) venom and their recombinant allergens (Api m1, Api m2, Api m3, Api m5, Api m10; Ves v1, Ves v5) and MUX F3; significant correlations are coloured in blue (r < 0.5 light blue, 0.5 ≤ r < 0.8 blue, r ≥ 0.8 dark blue); In participants with no history of anaphylactic reactions to Hymenoptera stings, most sIgE levels to the different allergens correlated moderately to each other, while in participants with a history of anaphylactic reactions sIgE levels to honey bee venom (i1) and wasp venom (i3) showed strong intercorrelations with their respective recombinant allergens but not with the allergens of the different species. ^a^ one missing case (N=202); * *P* < 0.05 ** *P* < 0.01. | | | | | | | | | | | | | |
